# Improved global 250 m 8-day NDVI and EVI products from 2000–2021 using the LSTM model

**DOI:** 10.1038/s41597-023-02695-x

**Published:** 2023-11-14

**Authors:** Changhao Xiong, Han Ma, Shunlin Liang, Tao He, Yufang Zhang, Guodong Zhang, Jianglei Xu

**Affiliations:** 1 School of Remote Sensing and Information Engineering, Wuhan University, Hubei, 430010 China; 2 JC STEM Lab of Quantitative Remote Sensing, Department of Geography, The University of Hong Kong, Hong Kong, China; 3 International Center for China Development Studies, The University of Hong Kong, Hong Kong, China

**Keywords:** Environmental sciences, Carbon cycle, Biogeochemistry

## Abstract

Satellite vegetation index (VI) products, such as normalized difference vegetation index (NDVI) and enhanced vegetation index (EVI), have been widely used. However, they are severely contaminated by clouds and other factors and provide false signals of the surface vegetation conditions. In this study, the new global seamless 250 m, eight-day NDVI and EVI products from 2000–2021 were developed from Moderate Resolution Imaging Spectroradiometer (MODIS) surface reflectance data using a long short-term memory (LSTM) neural network method. High-quality globally representative time series VI samples were constructed to train the model using a combination of the Savitzky-Golay filter (SG), Global LAnd Surface Satellite (GLASS) leaf area index (LAI) fitting and upper envelope methods. To evaluate the proposed method and the 250 m VI products, the MODIS VI product (MOD13Q1) was used for the inter-comparisons using four widely used VI reconstruction methods. Assuming that the MODIS VI data of high quality represents the true values, the root mean square error (RMSE) for NDVI and EVI generated by the LSTM model are 0.0734 and 0.0509, respectively.

## Background & Summary

Satellite products of vegetation indices (VIs) have been widely used for various purposes, including vegetation change monitoring^1,2^, vegetation phenology extraction^3,4^, terrestrial carbon circulation modelling^5,6^, dynamic environmental simulations^7,8^, and land coverage and change detection^9,10^.

Among them, the normalized difference vegetation index (NDVI) calculated from the near infrared band (NIR) and visible red band (RED) obtained by optical satellites is one of the most popular indices^11^.

Similar to the NDVI, the enhanced vegetation index (EVI) minimizes the canopy background variations and maintains its sensitivity under dense vegetation conditions. The EVI also uses the blue band (BLUE) to remove residual atmospheric contamination caused by smoke and thin sub-pixel clouds^12^.

However, for satellite-derived VI data, it is almost impossible to avoid adverse observation conditions such as clouds and sensor failure^12–15^, which greatly hinder the application of VI products. It is important to reconstruct the contaminated and missing data and obtain spatiotemporally continuous VI products^16,17^.

The VI reconstruction methods can be divided into two categories: the spatial and temporal based. Spatial based methods for the reconstruction of remote sensing images are most commonly used. Due to the high correlation between adjacent pixels, spatial interpolation according to the neighbouring effective pixel is effective under certain circumstances. Representative methods include the linear interpolation method^18^ and kriging interpolation^19^. However, when a spatial based reconstruction method is applied to remote sensing images with strong contamination or heterogeneity, its effectiveness will be greatly reduced^20^. Therefore, many reconstruction methods based on VI time series have been proposed. In the past few decades, dozens of time series VI smoothing methods^21^ have been developed. These methods can be divided into three categories according to their principles: (1) methods based on temporal information; (2) methods based on frequency information; and (3) hybrid methods. For the first category, some methods use sliding windows to filter information such as the best index slope extra (BISE) algorithm^22^, and the Savitzky-Golay (SG) filter^23^. Some use functions to fit local time series such as the asymmetric Gaussian (AG) function^24^, double logistic (DL) function^25^, or cubic spline polynomial^26^. Among the frequency information-based methods, the representative methods include the harmonic analysis of time series^27,28^ and the wavelet method^29^. In addition, there are many methods that use other principles^30^, such as the temporospatial filter (TSF)^31^ and the search and fill algorithm with moving offset method (SFA-MOM)^32^.

However, for traditional time-series based VI reconstruction methods, whether the method is based on temporal information or frequency information, the reconstructed VIs are significantly affected by the parameter settings. For specific situations in different regions, it is difficult to obtain consistent excellent results using traditional time-series based VI reconstruction methods^30,33,34^. VI reconstruction methods that rely on temporal and spatial information involve intricate algorithm designs and necessitate substantial computational resources. When employed in extensive regions, these methods tend to exhibit low computational efficiency^20^. Limited by the above conditions, the existing methods cannot efficiently generate spatiotemporally continuous global VI products. In recent years, with the increase in satellite data, deep learning neural networks have been widely used in remote sensing data processing, such as the classification of land types^35,36^, land surface biomass inversion^37^, and land surface temperature (LST) products^38,39^. The superb learning performance and computational efficiency of neural networks makes it possible to efficiently generate global products. The long-short term memory (LSTM) network can effectively process long-short temporal information and output the results^40^. Since the emergence of the LSTM, it has been used to predict and retrieve temporal information in many fields, such as computer vision, semantic recognition, acoustics, and remote sensing^41–44^. Recently, the version 6 leaf area index (LAI) product^45^ in the Global LAnd Surface Satellite (GLASS) products suite^46^ was produced using the LSTM, and high-quality results have been obtained^45^. The LSTM model has been proven to be effective in estimating continuous LAI data even when the high-quality surface reflectance input is absent for a long period. LAI and VI both function as indicators of plant growth, but they have distinct definitions and capture different aspects of vegetation dynamics. LAI represents the number of leaf layers per unit area, while VI, calculated directly from satellite reflectance, mainly reflects greenness and photosynthetically active biomass. These differences make each index suitable for specific applications and analyses. Owing to its direct relationship with vegetation structure, LAI is widely used in various climate and land surface models. On the other hand, NDVI is more user-friendly and can provide vegetation information for a wide range of applications, particularly among non-specialist users.

Therefore, this study is aimed to develop a LSTM based global VI reconstruction method similar to that of the GLASS V6 LAI. The core idea of this method is to create a globally representative sample based on LAI products and existing reconstruction methods. The high-quality VI time series of the sample pixels are reconstructed using a combination of LAI fitting, the upper envelope method, and the SG filter methods, and the results are used to train the LSTM model. The trained LSTM model produces spatiotemporally continuous global VI products.

## Methods

**Fig. 1:**
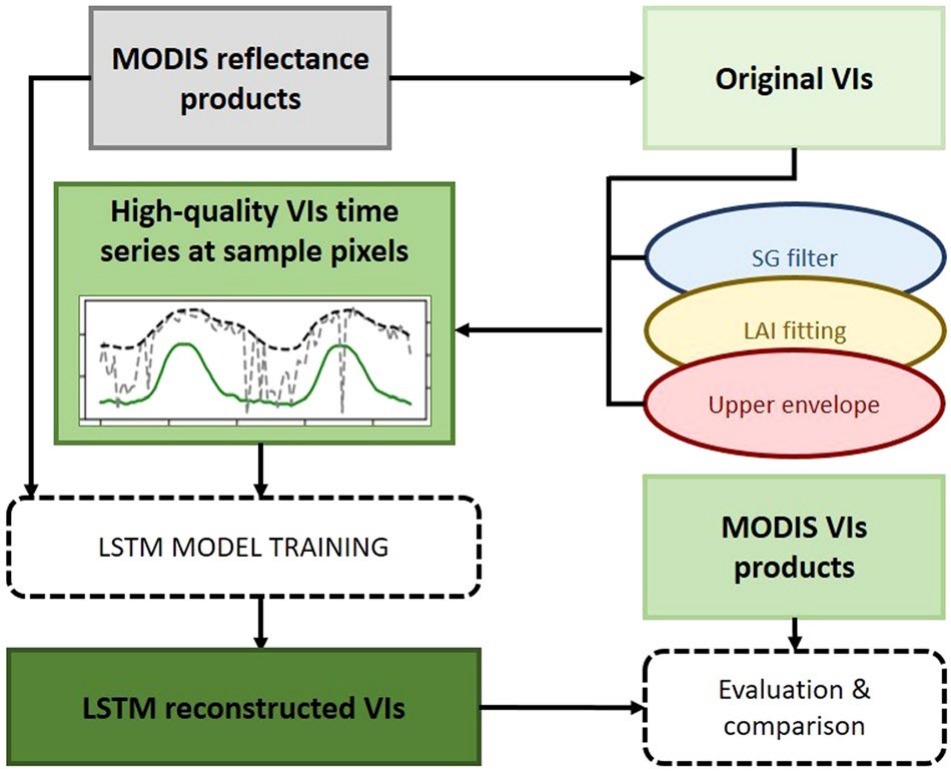
Overall Flowchart of the LSTM method.

In this study, a deep learning method was used to reconstruct the 250 m VI time series. The overall work flow of this research is shown in Fig. 1. First, the existing VI reconstruction methods and LAI products were used to reconstruct high-quality VI time series of the sample pixels. This step was the premise of the model training. Second, the reconstructed samples were used to train the model. The final step was product production and evaluation.

In this study, the NDVI and EVI were calculated as follows:

1 $$NDVI=\left(NIR-RED\right)/\left(NIR+RED\right)$$

2 $$EVI=2.5\ast \left(NIR-RED\right)/\left(NIR+6\ast RED-7.5\ast BLUE+1\right)$$

Four satellite data products were used. The GLASS LAI and MODIS surface reflectance products were used to create high-quality VI time series for sample pixels. The MODIS surface reflectance products were also used for training the LSTM model and producing the global NDVI and EVI products. The MODIS VI products were used for the inter-comparisons.

### Surface reflectance data

To obtain VI time series with a higher temporal resolution, MOD09Q1, and MOD09A1 version 6 products from 2014 to 2015 obtained from the MODIS sensor on-board the Terra satellite were used^47,48^. The MOD09Q1 product provides the surface spectral reflectance in bands 1 and 2 at a 250 m resolution and is corrected for atmospheric conditions such as gasses, aerosols, and Rayleigh scattering. Along with the two surface reflectance bands, a quality layer is also included^49^. For each pixel, a value is selected from all of the acquisitions within the 8-day composite on the basis of a high observation coverage, a low view angle, the absence of clouds or cloud shadows, and aerosol loading. In this study, the red band (250 m surface reflectance band 1 (620–670 nm)) and near infrared (NIR) band (250 m surface reflectance band 2 (841–876 nm)) from the product were used to calculate the NDVI.

When calculating the EVI, the blue band (500 m surface reflectance band 3 (459–479 nm)) provided by the MOD09A1 was used. This band was also used to calculate the EVI in the MODIS VI product (MOD13Q1). In addition, the same calculation formulas and parameter settings were used because MOD13Q1 has been widely used. This product was also used as a contrastable object in the evaluation.

When the VIs were calculated, the basic processing of the results was conducted. When the value of the surface reflectance were negative and the solar zenith angle was greater than 85°, the calculated VI values were invalid, and the value was set to −0.2^48^. The red band, near infrared band, and blue band were also used as input data for the LSTM model training and calculation.

### LAI data

The GLASS V6 LAI data were used to fit the original VI time series, which is one of the methods used to generate high-quality VI time series of the sample pixels^45^. GLASS V6 LAI is a globally seamless spatiotemporal LAI product, derived from three distinct LAI datasets using a deep learning model^45^. This unique approach allows it to assimilate data from multiple sensors and effectively correct for weather-induced degradation. Compared with the previous iteration, GLASS V6 LAI delivers heightened reliability and consistency in depicting vegetation dynamics, even under the presence of cloud cover or adverse meteorological conditions. In this study, we leverage the attributes of GLASS LAI during the construction of vegetation index samples. Our objective in incorporating GLASS LAI data is to mitigate the prolonged impact of cloud cover and other weather-related factors that could potentially compromise the quality of NDVI data. The GLASS V6 LAI dataset was downloaded from http://www.glass.umd.edu/LAI/MODIS/250 m/ (last access: 3 November 2022, Ma, and Liang, 2022d).

### VIs data

The MOD13Q1 product provides VIs data with a spatial resolution of 250 m and a temporal resolution of 16 days. It contains two vegetation index layers^50^. The first is the NDVI, and the second is the EVI, which minimizes the canopy background variations and maintains its sensitivity under dense vegetation conditions. This product provides a detailed quality assurance (QA) layer and a summary QA layer to measure the availability and accuracy of the vegetation index layers. The QA indicates if a pixel is affected by cloud contamination or covered by snow/ice. In the evaluation, the VIs and related QA information provided by MOD13Q1 were used to compare the results of the LSTM method with those of other methods.

### Global representative samples

Training samples are very important in deep learning research, and they directly determine the scope of the application of the model and its universality^51^. In previous studies, 52997 sample pixels distributed around the world were selected^45^. They were selected based on global time series LAI clustering analysis and the least difference criterion, as well as by assuming that the LAI values of three widely used products with the lowest mean square errors (MSEs) represented the true values of specific pixels. These sample pixels can represent different vegetation types, different observation qualities, and different geographical locations around the world at the same time, which meets the needs of reconstructing VI time series.

The LSTM’s superb learning performance allowed us to efficiently obtain results with a high quality similar to the high-quality VI time series of the sample pixels^52^. Thus, other auxiliary data can be introduced to ensure the quality. During production, the LSTM does not need to use the auxiliary data, which reduces the demand for data and computing resources. For the VI time series, the pollution caused by clouds and snow is the main cause of the low-quality data^12,13,15^. Although there were low-quality data with high values, this type of phenomenon was relatively scarce in the NDVI time series. For the EVI time series, a simple threshold method was used to remove these data. In this study, it was assumed that the data with higher values in the NDVI time series and the pre-processed EVI time series were of high quality, so the high-quality VI time series were similar to the upper envelope of the original VI time series. In addition, the VIs were indices that represent the vegetation status, and many studies have also indicated that VIs are significantly proportional to the LAI^53–55^. Therefore, it was assumed that the overall patterns of increase or decrease of the high-quality VI values throughout the seasons will follow a similar pattern as the changes in LAI values.

In order to make the results consistent with the above assumptions, four steps were used to create high-quality VI time series of the sample pixels.

The first step was to conduct the SG filtering process to roughly remove the contaminated values. The SG filtering can be interpreted as a weighted moving average window, and the weighting was given as a polynomial of the window width. It was designed to fit the upper envelope and to describe the changing patterns of the parameter through an iteration-process. According to the user guide provided by TIMESAT^24^, the half-width of the smoothing window of the SG filter was set to 12 (number of values per year/4), and the number of iterations was set to 2. The specific method was that when there was a value higher or lower than the SG filtering result in the original time series and it exceeded the threshold, the value was labelled as an invalid value. In this study, the threshold was set to 0.2 for the EVI time series and 0.4 for the NDVI time series, and the invalid value was set to −0.2.

The second step was the NDVI-LAI fitting. The GLASS LAI data were introduced to fit the VI values that had been processed using the SG filter. For each VIs time series, an exponential function was used to establish the relationship between the LAI value and the high-quality VI value using the least squares method and to obtain the new VI time series returned by the LAI time series. The function can be expressed as

3 $$VI=c-{e}^{(a\ast LAI+b)}$$

Equation (3) was used because the VIs were saturated when the LAI value was high. This saturation phenomenon not only led to nonlinearity of the regression, but also the inconsistencies between the trend patterns of the VIs and LAI at high values. In the third step, the upper envelope method was used to effectively solve this problem.

As an auxiliary method, the principle of the upper envelope method is very simple. It can roughly be summarized as follows. If a value in the time series is higher than the previous and next value, this value is determined to be an envelope value. Then, the value that is not selected will be linearly interpolated according to the previous and next envelope value. A new time series is synthesized by replacing the value in the results of the NDVI-LAI fitting method with the corresponding higher value in the results of the upper envelope method. This can be expressed as follows:

4 $$NDV{I}_{syn}=max\left(NDV{I}_{fitting},serie{s}_{env}\right)$$

*NDVI*_*fitting*_ is the NDVI time series returned by the LAI time series. *series*_*env*_ is the upper envelope time series obtained using the third step. Finally, the SG filtering method is used to process the synthetic times series to obtain the final high-quality VI time series of the sample pixels.

Most of the samples in this study were processed using the above steps, but there were two exceptions.

First, for the time series with a minimum LAI of greater than 5 in two years, due to the severe saturation phenomenon, the LAI fitting fails, and there are even negative correlations between the VIs and the LAI. In order to deal with this phenomenon, *a* in Eq. (3) is limited to being greater than zero, and the data in the time series with higher values than the result of the first fitting are used for the second fitting. The remaining steps are consistent with the above descriptions.

Second, for pixels located at high latitudes that are covered by snow and ice for a long time in winter, their time series data are also inconsistent with the trend of the LAI because of the extremely low NDVI values caused by the snow and ice. These extremely low NDVI values are consistent with the actual situation of the surface, so even if they have different trends from the LAI, they should be retained. Equation (5) was used to deal with the above problem.

5 $$NDVI=\left(NDV{I}_{ori}+0.2\right)/0.4\ast NDV{I}_{syn}+\left(1-\left(NDV{I}_{ori}+0.2\right)/0.4\right)\ast NDV{I}_{ori}$$

SG filtering is applied to the original NDVI time series. For values of less than 0.2 in the filtering result, Eq. (5) is used for the processing. *ndvi*_*ori*_ is the corresponding original NDVI. *ndvi*_*syn*_ is the corresponding synthetic NDVI mentioned above. Note that the coefficients in Eq. 5 for processing sample pixels in ice and snow regions are determined through empirical analysis and experimentation. This step enables the reconstructed NDVI to retain the low value characteristics caused by long-term snow and ice cover.

### LSTM deep learning model training

The LSTM network is an improved structure of a recurrent neural network (RNN), which is a feed forward network with a feedback loop and internal memory^56^. When using low-quality data for the training process, the RNN can use its own structure to deal with these shortcomings^57^. As an improved structure of the RNN, the LSTM network performs better regarding the problems of exploding and vanishing gradients^58^. More importantly, the LSTM has a long-term memory compared to the RNN, with an input gate, output gate, and forget gate in each layer of the network. More details have been provided by this study^45^.

#### Datasets for model training

For training the LSTM model, the surface reflectance data for 2014 and 2015 from MOD09A1 and MOD09Q1 were collected. The high-quality VI time series created were considered to be the target of the model training. While training, the sample pixels were randomly divided into three parts. Eighty percent of the sample pixels were used to train the model, 10% were used to optimize the model, and 10% were used to verify and evaluate the quality of the model and results.

#### Model training

The deep learning model developed in this study is based on Python3.8 (PyTorch). This model comprises an input layer, a three-layer LSTM with eight hidden nodes each, an activation function layer, and an output layer. Within the activation function layer, Gaussian error linear units (GELU) are employed as the activation function, known for their high-performance in neural networks and their capacity to enhance the network’s ability to fit nonlinear relationships. Thanks to the LSTM’s inherent characteristics, the original surface reflectance data can be directly used for model training without requiring additional pre-processing steps. The model undergoes iteration and optimization with the Adam optimizer, with the learning rate dynamically adjusted between 0.0001 and 0.00000001 using the lr_scheduler function provided by PyTorch. This method causes the learning rate drop from the maximum value to the minimum value following the morphology of the sinusoidal function, which makes the optimization more accurate. The three bands used in the VIs formulas are used as the input data (i.e., the red band, near infrared band (b1 and b2 from MOD09Q1), and blue band (b3 from MOD09A1)). In order to improve the training efficiency, the time span of the sample is shortened as much as possible. Two years of time series data contain both intact vegetation growth cycles and information between the previous year and the next year. Therefore, two years is considered the most appropriate time span. Data from 2014 to 2015 were used in this study.

### NDVI and EVI products evaluation

The obtained VI time series were evaluated based on three aspects. Due to the lack of true observations of VIs, in recent years, most studies complete the quantitative part of the evaluation by generating a reference time series. The mean values of high-quality observations acquired on the same date in the long time series are calculated, and these mean values are used to generate high-quality a reference time series. This method ignores the case where the pixels change during the long period of time. Moreover, the reference time series generated does not exist in practice, and even when noise is artificially added to this reference time series, LSTM cannot learn such a feature during training. Thus, this method was not applicable in this study. In this study, we quantitatively and qualitatively evaluated the LSTM products using MOD13Q1 and by visually analysing the time series and spatial characteristics.

MOD13Q1 was calculated from MOD09Q1 using the constrained view angle-maximum value composite method (CV-MVC) to obtain VI values that were considered to have the highest quality within 16 days. MOD13Q1 also provides a layer that describes the data quality (i.e., summaryQA), in which the best and useful data are set to one and zero, while the cloud-contaminated and ice-covered data are set to 2 and 3. It was assumed that the reconstructed VIs with a better quality should be more consistent with the best and useful data in MOD13Q1.

In order to compare the LSTM method and the other reconstruction methods, in this study, four commonly used VI reconstruction methods, namely, the SG filter (SG), double logistic (DL), asymmetric Gaussian (AG), and Whittaker smoother (WT) methods were applied. Among them, the SG, DL, and AG are provided by TIMESAT version 3.3^24^. The WT can be regarded as the penalized least squares method, which puts a fidelity penalty on the roughness of the smooth curve^59^. According to the user guide provided by TIMESAT, the half-width of the smoothing window of the SG was set to 12 (number of values per year/4), and the number of iterations was set to 2. For the DL and AG, the seasonality parameter was set to 0.5. For the WT, according to previous research^60^, the default smoothing parameter was set to 2.

The root mean square error (RMSE) and coefficient of determination (R^2^) were used to evaluate the performances of the five methods. The RMSE reflects the fidelity of the reconstruction results compared to the high-quality data in MOD13Q1. The R^2^ value is between 0 and 1, and its value reflects the adequacy of the independent variable’s interpretation of the dependent variable. R^2^ is one of the most common indexes used to evaluate regression models. These two parameters can be calculated as follows:

6 $$RMSE=\sqrt{{\sum }_{i=1}^{n}\left({y}_{resul{t}_{i}}-{y}_{goo{d}_{i}}\right)}$$

7 $${R}^{2}=\frac{{\sum }_{i=1}^{n}{\left({y}_{resul{t}_{i}}-{y}_{average}\right)}^{2}}{{\sum }_{i=1}^{n}{\left({y}_{o{b}_{i}}-{y}_{average}\right)}^{2}}$$

n is the total number of observations with good quality. ${y}_{o{b}_{i}}$ is the value of an observation, ${y}_{resul{t}_{i}}$ is the value of the corresponding reconstruction result, and *y*_*average*_ is the average value of the observations of high-quality data in MOD13Q1.

Two other parameters were used as a reference to evaluate the results^61^. One was the upper envelope rate, which is defined as the ratio of the reconstruction results with values lower than the original observations. For the NDVI, better reconstruction results should have a lower envelope rate. The other was the distance between the reconstruction results and the original observations, which is defined as the average of the absolute value of the difference between all of the reconstruction results and the original observations. It measures the fidelity of the reconstructed results compared to the original observations, and good reconstruction results should not have large elevation distance values.

The above parameters were calculated using independent sample pixels on a global scale reserved for evaluation. In addition to quantitative evaluation, in this study, qualitative evaluation was also conducted. This was performed at the selected sample pixels and in the selected spatial area.

### Data production

The production of the products continues to be based on PyTorch. By inputting the near-infrared band and red band of MOD09Q1 and the blue band of MOD09A1 into the trained LSTM model and performing calculations, time-series results can be obtained. Then, the results are organized into the same projection and tiling as the MODIS reflectance product through matrix transformation. These products use the same land-sea mask as GLASS V6 LAI, and the ocean part will be assigned invalid values.

## Data Records

The improved global NDVI and EVI products from 2000–2021 are freely available from figshare. The 250 m 8-day GLASS VIs products for the first day in 2000 is freely available at 10.6084/m9.figshare.22220050, 10.6084/m9.figshare.22220125^62,63^. We have also aggregated it to coarser resolutions(0.05° 8d, 0.1° per month, and 0.25° per month; 10.6084/m9.figshare.22267048^64^). The 250 m data are in the sinusoidal projection, whereas the 0.05, 0.1, and 0.25 data are in the geographic latitude and longitude coordinate system. The data files are provided in Hierarchical Data Format-Earth Observing Systems (HDF-EOS) format. The 250-m 8-day NDVI files are named in the following format: “GLASS13D01.V10.AYYYYDDD.hHHvVV.yyyyddd.hdf”, where “GLASS13D01”, “V10”, “YYYY”, “DDD”, “HH” and “VV” denote the product name, version number, year, Julian day of the year (doy), and MODIS tile ID, respectively. The lowercase letters “yyyyddd” represent the year and doy of the processing date. For the EVI files, the product name is “GLASS14D01”. Additional information, such as the scale factor and value range, is stored in the files.

## Technical Validation

In this section, the LSTM-reconstructed VIs are displayed on the global scale. Quantitatively comparison and analysis of the performance of the LSTM method and the other reconstruction methods are conducted based on MOD13Q1. In terms of the qualitative analysis, the reconstruction methods are compared in both space and time.

### Global reconstruction results

**Fig. 2:**
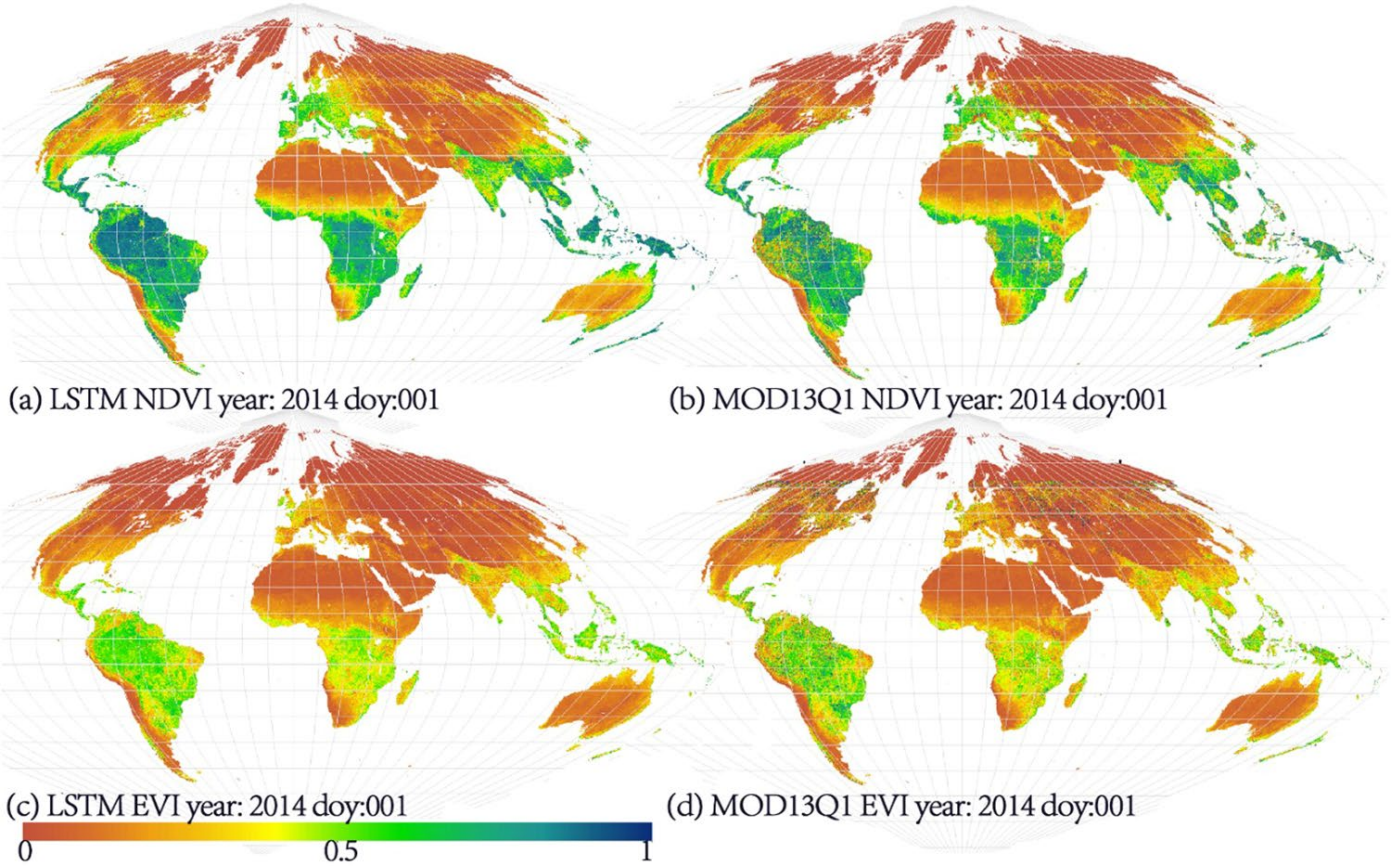
Global VIs of the LSTM method and MOD13Q1; (**a**) LSTM NDVI, year: 2014 DOY:001; (**b**) MOD13Q1 NDVI, year:2014 DOY:001; (**c**) LSTM EVI, year:2014 DOY:001; (**d**) MOD13Q1 EVI, year:2014 DOY:001.

Figure 2 shows the global reconstructed VIs (VI reconstruction results) and the MOD13Q1 product. As can be seen, the spatial distribution of the LSTM-reconstructed VIs is the same as that of the MOD13Q1 product.

**Fig. 3:**
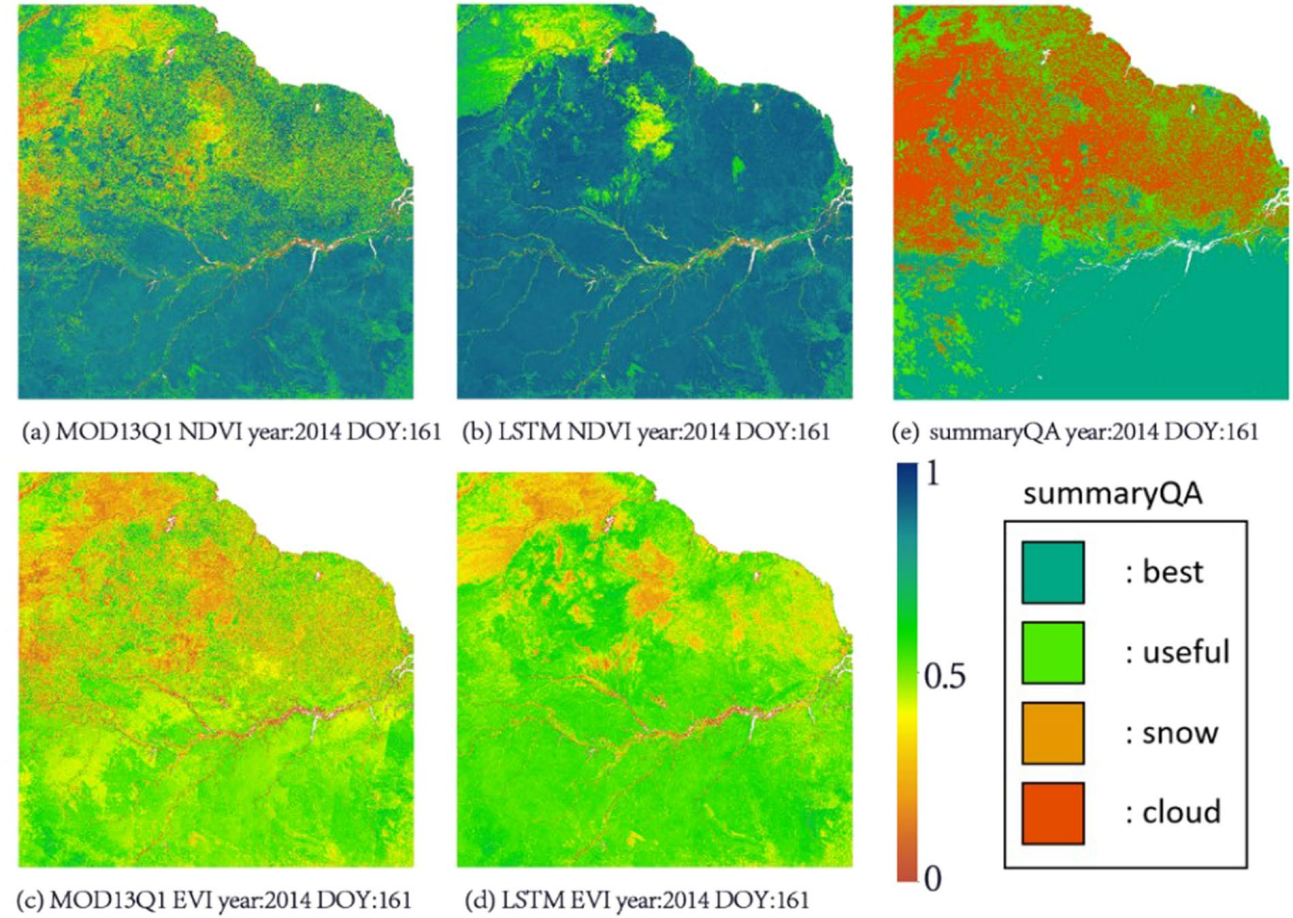
Northeast South America VIs of the LSTM method and MOD13Q1; (**a**) MOD13Q1 NDVI, year:2014 DOY:161; (**b**) LSTM NDVI, year:2014 DOY:161; (**c**) MOD13Q1 EVI, year:2014 DOY:161; (**d**) LSTM EVI, year:2014 DOY:161; (**e**) MOD13Q1 summaryQA, year:2014 DOY:161.

**Fig. 4:**
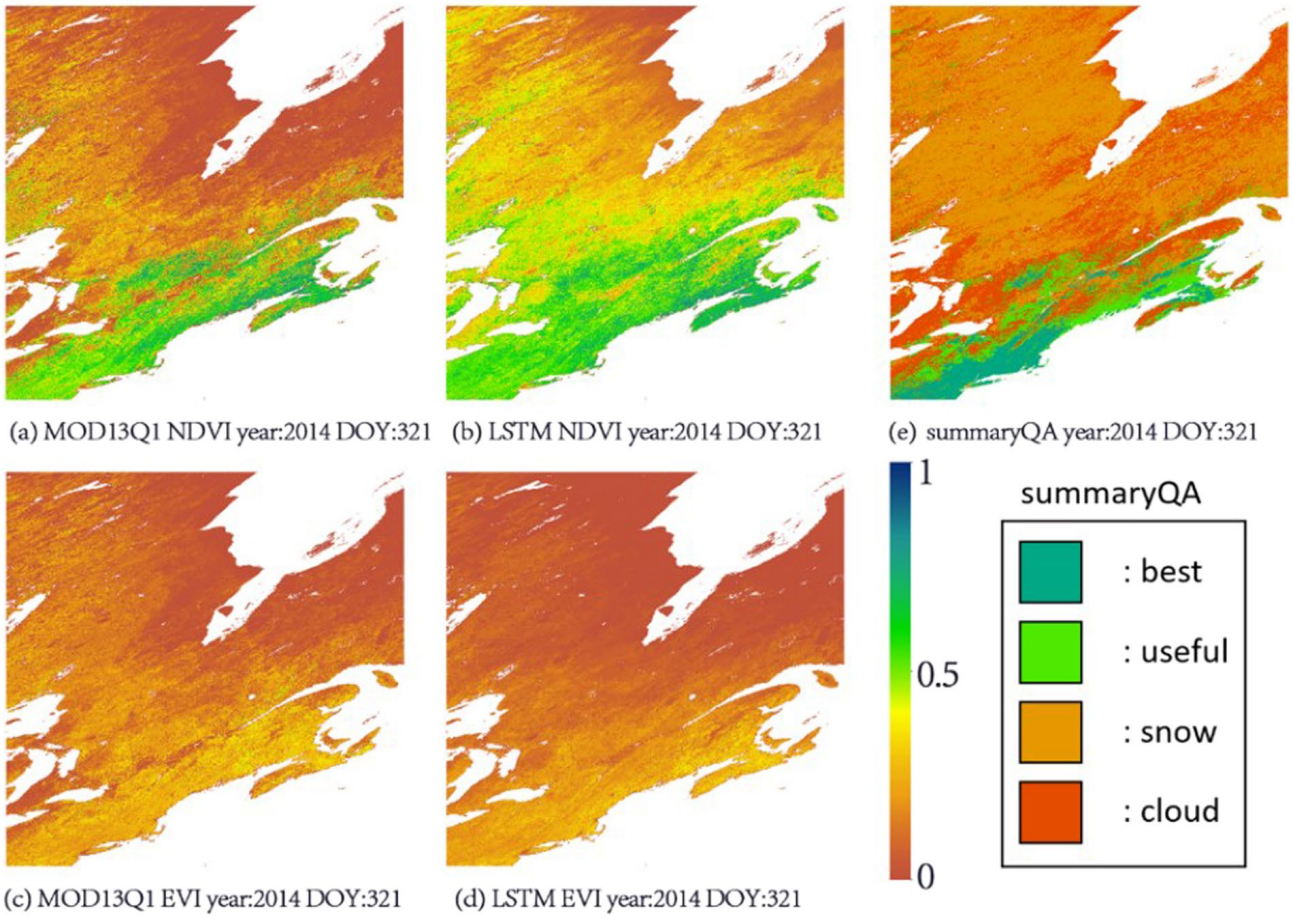
Northeast Canada VIs of the LSTM method and MOD13Q1; (**a**) MOD13Q1 NDVI, year:2014 DOY:321; (**b**) LSTM NDVI, year:2014 DOY:321; (**c**) MOD13Q1 EVI, year:2014 DOY:321; (**d**) LSTM EVI, year:2014 DOY:321; (**e**) MOD13Q1 summaryQA, year:2014 DOY:321.

In the calculation of the VIs, in the observation process, clouds and snow often leads to low-quality data. The global distributions of clouds and snow has very significant spatial and temporal characteristics. According to a previous study, the tropical regions near the equator and at mid-high latitudes have the highest proportion of low-quality observations^65^. In these areas, the reconstruction of VIs is more difficult. Therefore, the north-eastern part of South America and Canada were selected for detailed analysis. The LSTM-reconstructed VIs and MOD13Q1 for these areas are shown in Figs. 3, 4.

In Figs. 3, 4, it can be clearly seen that when the observations were affected by clouds and/or snow, the spatial distributions of the VIs became very uneven. Even though MOD13Q1 calculates relatively high-quality VIs by reducing the time resolution to 16 days and using the CV-MVC algorithm, in the summaryQA layer in MOD13Q1, when the quality of the observation is low, the NDVI will have low values in the area with clouds and/or snow, while the EVI will contain a large amount of noise in the corresponding area. The above phenomena are consistent with our predictions, and the LSTM reconstruction method also effectively eliminates these problems.

### Quantitative evaluation

**Fig. 5:**
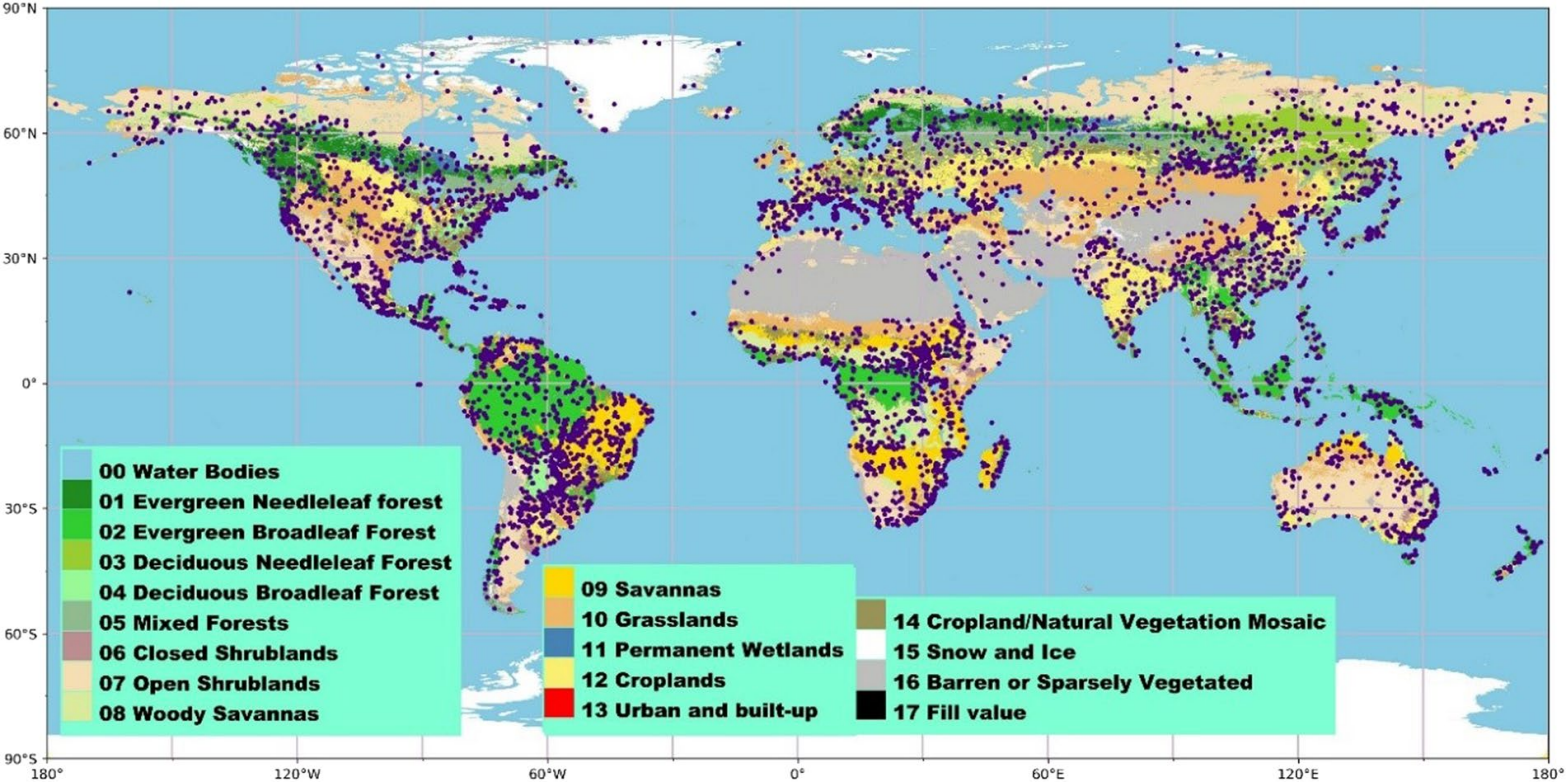
global distribution and land cover type of the selected evaluation pixels.

Figure 5 shows the global distribution of the selected evaluation pixels, and the results are presented in Table 1.

**Table 1:** Evaluation of the LSTM method compared with other methods.

MOD13Q1 QA RMSE R^2^ NDVI								
	all		40°N-60°N		10°S-10°N		env_rate	distance
	RMSE	R^2^	RMSE	R^2^	RMSE	R^2^		
LSTM	0.0734	0.8914	0.0788	0.8616	0.0799	0.8345	0.13	0.13
SG	0.0808	0.8682	0.1052	0.7533	0.0824	0.8239	0.4	0.11
AG	0.082	0.8643	0.1048	0.7551	0.0822	0.8247	0.38	0.11
DL	0.0801	0.8707	0.1035	0.7613	0.0799	0.8341	0.38	0.11
WT	0.117	0.7237	0.1602	0.4278	0.1181	0.6378	0.55	0.1
**MOD13Q1 QA RMSE R**^**2**^ **EVI**								
LSTM	0.0509	0.8926	0.0499	0.8788	0.0636	0.8186	0.44	0.06
SG	0.0605	0.8479	0.0573	0.8406	0.0745	0.7509	0.33	0.05
AG	0.0659	0.8197	0.059	0.8309	0.0831	0.69	0.32	0.06
DL	0.0639	0.8302	0.0586	0.8332	0.0794	0.717	0.31	0.06
WT	0.0624	0.838	0.0563	0.8461	0.0811	0.7048	0.5	0.04

A total of 5301 evaluation pixels are shown in Fig. 5, which are mainly distributed on the surfaces covered by vegetation, and the vegetation types are diverse. Such sample pixels meet the requirements for our research.

Table 1 shows the good performance of the LSTM method. For the different latitudes and VIs, the LSTM method achieved the minimum RMSE and maximum R^2^, demonstrating that the LSTM-reconstructed VIs were the most consistent with the high-quality observation data of the MOD13Q1 product. For the other two parameters, the upper envelope rate and the distance, the LSTM method achieved the expected effect. For the reconstruction of the NDVI, the LSTM method had the lowest upper envelope rate, indicating that its results were closest to the upper envelope of the original time series. The distance results obtained for all of the methods were relatively similar, indicating that the LSTM-reconstructed VIs have similar fidelities to those of the other methods. In detail, among the four methods, the SG method usually achieved the best results, except for the LSTM method. In addition, the WT method has been widely used to reconstruct the EVI. Table 1 shows that the WT method achieved better results than the SG filter in reconstructing the EVI at middle and high latitudes. Among the methods, the SG and WT methods were second only to the LSTM method. In the subsequent qualitative evaluation, we will compare these two methods to the LSTM method.

### Qualitative evaluation

Due to their superior performances compared with the other methods, in this section we focus on comparing the SG and WT methods with the LSTM method. The evaluation will be carried out from two aspects: the VI time series curves of representative pixels and the spatial distributions of the reconstruction results. The representative pixels and areas selected for display are guided by last section, that is, the pixels and areas with large differences in the results of the different reconstruction methods are displayed to highlight the advantages and disadvantages of these methods.

#### Evaluation based on the curves of the representative pixels

This section displays the VI time series curves with the greatest differences according to the differences in the reconstruction results of the different methods. Curves with similar shapes are not displayed repeatedly in the following figures.

**Fig. 6:**
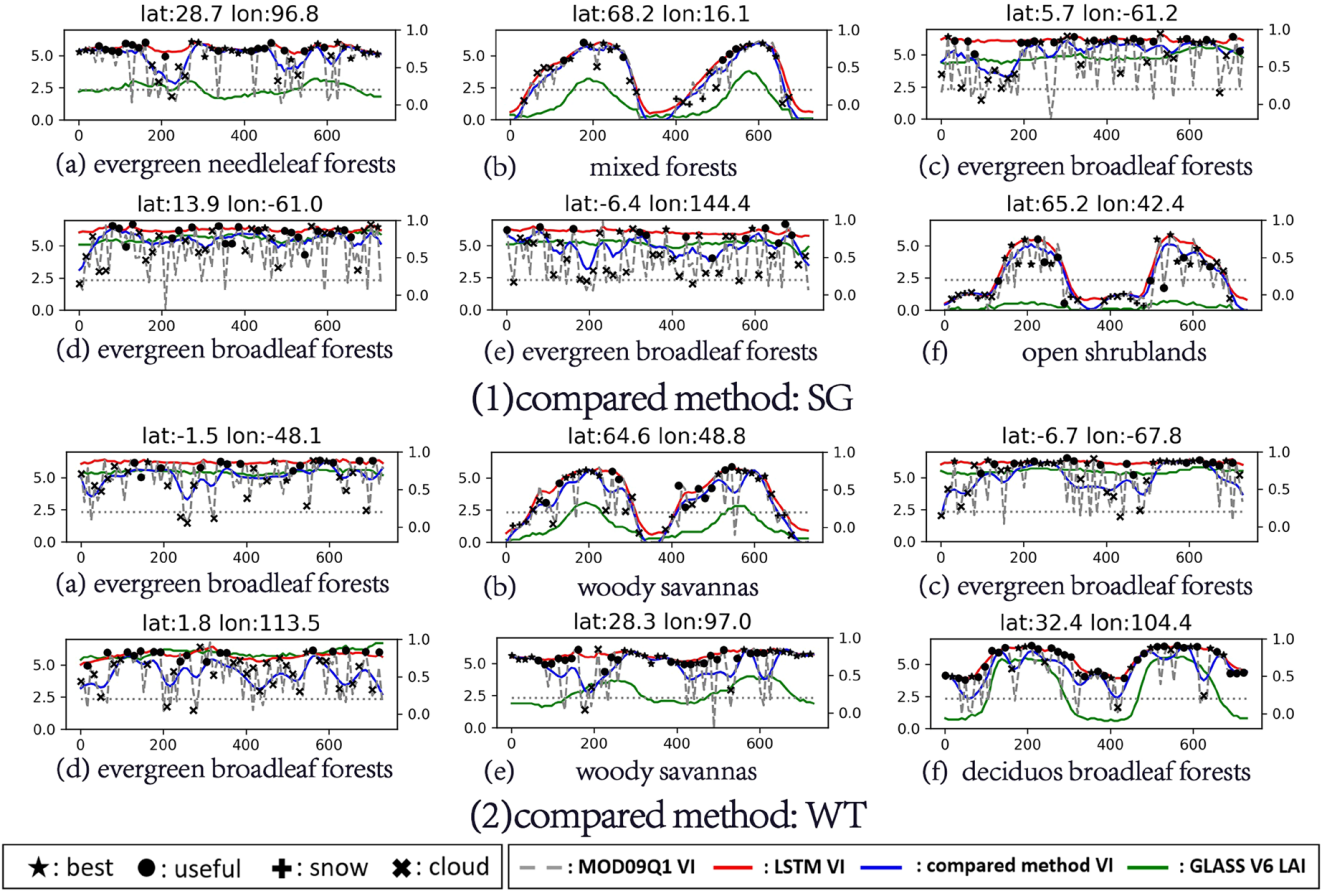
Curves with large differences in the representative pixels. Reconstruction of the NDVI for the LSTM method compared with (1) SG, and (2) WT.

Figure 6 shows the performances of the three methods in reconstructing the NDVI. Compared with the LSTM method, the reconstruction results of the WT and SG methods are limited in the following cases. First, in the tropical area, due to the influences of the large number of clouds and the poor observation conditions, there are no high-quality data for a long time. This leads to underestimation of the reconstruction results of the SG and WT, and almost none of the time filtering methods can solve this problem. Second, in the middle and high latitudes, due to the influences of snow and the observation angle, there are a large number of invalid data in the original surface reflectance data. In this case, the reconstruction results of the SG and WT are inevitably affected by the filling value, and the quality of the reconstruction results is highly related to the filling method. In this experiment, the reconstruction results of the SG and WT are significantly underestimated because the filling value was set to – 0.2. In contrast, due to the introduction of the LAI information, the LSTM method can effectively eliminate the adverse effects and reconstruct a high-quality NDVI time series when a large number of low-quality observations and invalid values exist for a long time.

**Fig. 7:**
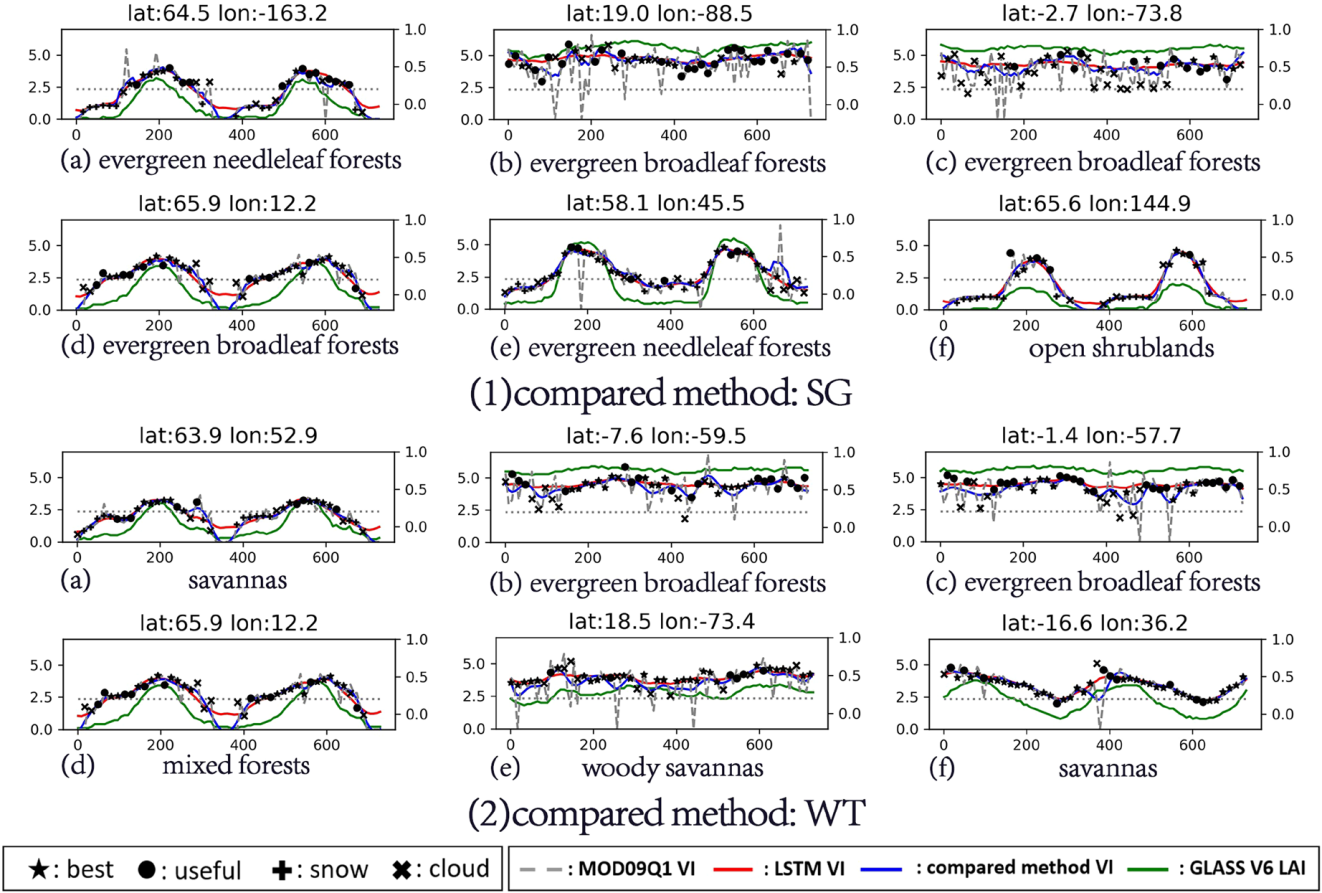
Curves with large differences in the representative pixels. Reconstruction of the EVI for the LSTM method compared with (1) SG, and (2) WT.

For the EVI in Fig. 7, the situation is similar to that for the NDVI. In the two cases mentioned above, the low-quality EVI data often appear as noise with high or low values. This creates challenges for the LSTM method and the other methods because the goal of reconstruction is no longer to reconstruct the upper envelope curve. Even so, for the LSTM method, which introduces the LAI, its results also recover the variation trend of the EVI with the LAI. For the other two methods, in addition to the problems encountered when reconstructing the NDVI, when reconstructing the EVI, the low-quality data with high values have a great impact on the final results, thus reducing the quality of the results.

### Evaluation based on the spatial distribution

In this section, the experimental area corresponds to 400 × 400 pixels in a tile of the MOD09A1 product. The selected areas are the areas located in the centers of tile h28v06 and tile h10v08. The original VIs and LSTM-reconstructed VIs corresponding to the first half of 2014 in this region are displayed. The EVI reconstruction results obtained using the WT method and the NDVI reconstruction results obtained using the SG method are also displayed. According to the quality file provided by MOD09Q1, the cloudy portion of the original vegetation indices is set to −1.

**Fig. 8:**
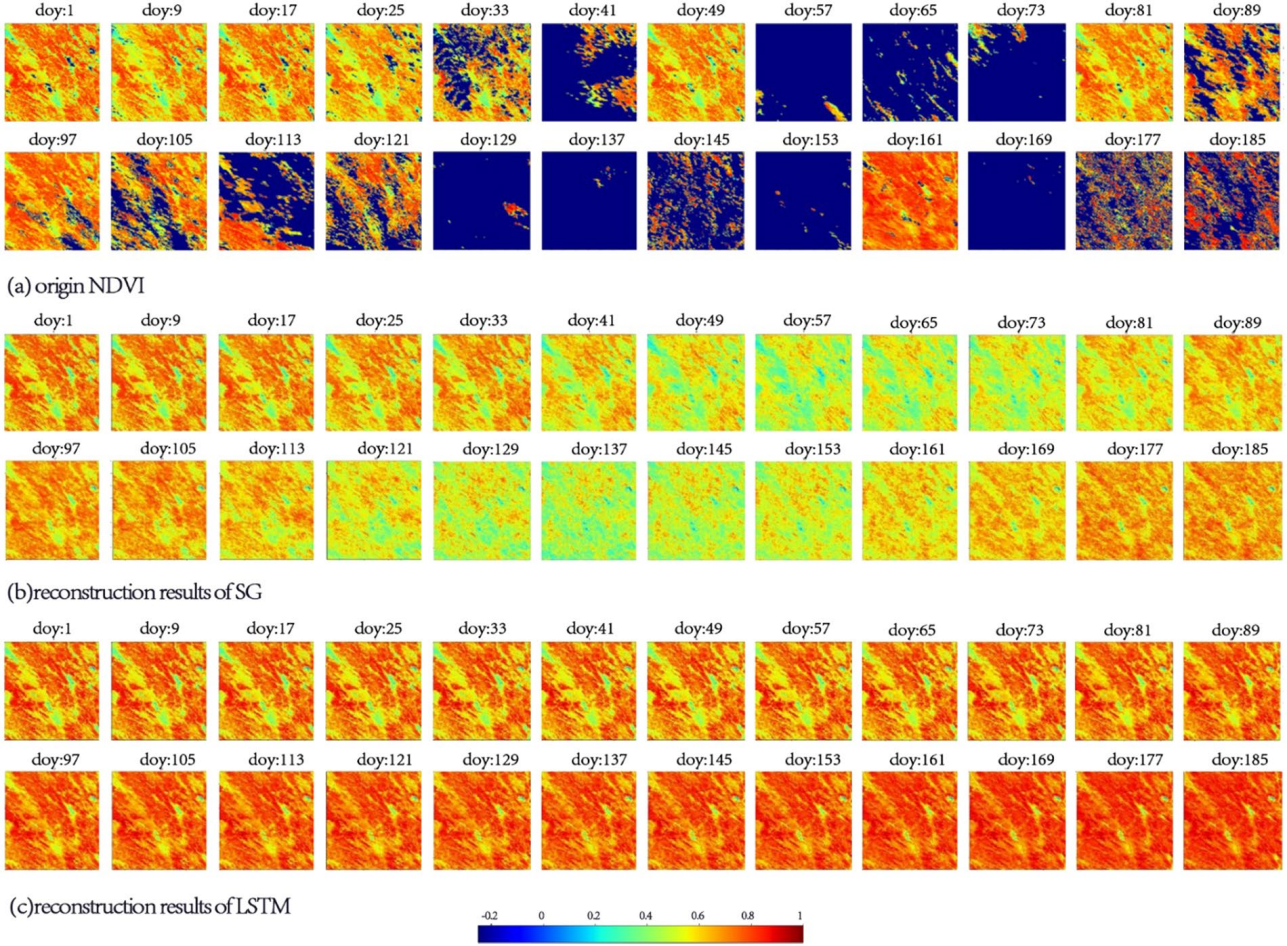
Spatial distribution of different results in 2014, area: 100 km*100 km, central pixel: 25.00°N, 115.86°E, VI: NDVI, DOY: 1–185; (**a**) original NDVI, (**b**) reconstruction results of SG, and (**c**) reconstruction results of LSTM.

**Fig. 9:**
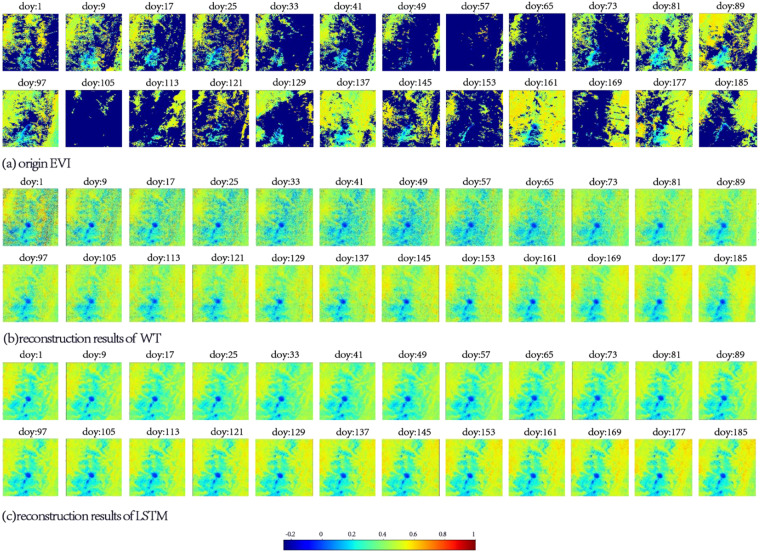
Spatial distribution of different results in 2014, area: 100 km*100 km, central pixel: 5.00°N 75.28°W, VI: EVI, DOY: 1–185; (**a**) origin EVI, (**b**) reconstruction results of WT, (**c**) reconstruction results of LSTM.

Figures 8, 9 show that the SG and WT methods have a certain effect on the reconstruction of the VIs. However, consistent with the conclusions in the previous section, when the observations are polluted by continuous clouds, the quality of the SG and WT results cannot meet the requirements. The corresponding area in Fig. 8 is located in southeastern China and has a subtropical monsoon climate. This makes the region rich in precipitation in spring and summer, which manifests as the large number of low value areas in Fig. 8(a). On doy 57 and 121, there were two consecutive low-quality observation periods of more than 21 days in the region.

In Fig. 9, the area shown is located in the northeastern part of South America. The observations in this area were disturbed by perennial clouds and precipitation, which is reflected in the EVI as a large amount of discrete noise. Figure 9b shows that the WT method cannot effectively remove the noise pixels. For the first 73 days of the year, low-quality values still exist in the results of the WT method.

## Usage Notes

This study presents a method for reconstructing global 250 m NDVI and EVI using LSTM networks and MODIS surface reflectance data. Specifically, high-quality VI time series were generated for 52997 pixels by leveraging MODIS surface reflectance data from 2014–2015 and the GLASS V6 Leaf Area Index (LAI). Due to methodological assumptions, this product theoretically exhibits the highest quality in areas covered by vegetation. In areas devoid of vegetation, such as deserts or urban zones, the LAI value typically remains extremely low or approaches zero. GLASS LAI offers data for these regions, as it can furnish insights into the presence of sparse vegetation and the dynamics of vegetation cover changes over time. Moreover, the temporal curve of this product generally aligns with the LAI trend while preserving its inherent characteristics. In addition, the low observation quality in high latitude regions may affect the product to some extent, but this only occurs at the beginning and end of the year and the impact is minimal.

## Data Availability

The Python codes for generating and processing data and be accessed through GitHub (https://github.com/Xiongkovsky/glass_vis_lstm_code).
